# Influence of Heat Treatment on Cyclic Response of Nickel-Based Superalloy Inconel 718 up to Very-High Cycle Regime

**DOI:** 10.3390/ma13235358

**Published:** 2020-11-26

**Authors:** Mengxiong Zhao, Zhenhua Zhao, Lulu Liu, Gang Luo, Wei Chen

**Affiliations:** 1 Aero-Engine Thermal Environment and Structure Key Laboratory of Ministry of Industry and Information Technology, College of Energy and Power Engineering, Nanjing University of Aeronautics and Astronautics, Nanjing 210016, China; zhaozhenhua@nuaa.edu.cn (Z.Z.); liululu@nuaa.edu.cn (L.L.); cepedzb@nuaa.edu.cn (G.L.); chenwei@nuaa.edu.cn (W.C.); 2 Laboratoire Energétique Mécanique Electromagnétisme, EA4416, Université Paris X Nanterre, 50 Rue de Sèvres, 92410 Ville d’Avray, France

**Keywords:** heat treatment, microstructure, very high cycle fatigue, ultrasonic, Inconel 718, self-heating

## Abstract

Cyclic response and fatigue behavior are sensitive to the microstructure of material induced by heat treatment. In this paper, three sets of high-temperature superalloy Inconel 718 with different heat treatment, namely annealed, aged, and directly aged high quality (DAHQ), are compared. Difference in grain size distribution, phase, and precipitate, etc., were investigated using an optical camera and scanning electron microscopy. Yield and ultimate strength were found to increase obviously after aging heat treatment. Self-heating phenomenon at 20 kHz was attenuated as grain size decreased. There was a transition from cyclic hardening to softening. Very-high cycle fatigue (VHCF) behavior of Inconel 718 was tested using an ultrasonic fatigue device. Crack initiation duration occupied greater than 99% of the total fatigue life. It concluded that average grain size influences VHCF strength and crack initiation mechanism, and that self-heating phenomenon is not a decisive factor on VHCF strength for Inconel 718.

## 1. Introduction

Very-high cycle fatigue (VHCF) is defined as a fatigue after 10^7^ cycles [1]. Most of traditional fatigue test machines that work at several hundred Hz are not suitable for experimental investigation in the VHCF range. The ultrasonic fatigue test method has been used to explore fatigue life [2] and mechanisms [3] at the very-high cycle domain, and for investigating threshold crack propagation behavior [4]. Bathias [5] found that microstructure instability is related to a heating dissipation, with an increase of the temperature able to explain the frequency effect in some metals. Crack initiation prefers from inclusions if the load ratio (***R***) is high. Porosities can be initiated in competition with inclusions when the load ratio is low, particularly in tension compression. Pineau [6] indicated that the microstructure instability could produce large dispersion and uncertainty in fatigue life and microcrack nucleation mechanism, and then results in the shape change of the ***S-N*** curve.

High-temperature superalloy Inconel 718 is widely used in the aeronautic applications, due to its high resistance to corrosion, oxidation, thermal creep deformation, and its high mechanical strength at elevated temperatures up to 700 °C [7]. The strength of superalloy Inconel 718 comes from coherent solid-state precipitates, which comprise small amounts of γ′, but mostly γ″, and produce coherency strains in the γ face center cubic (FCC) matrix [8,9]. Non-strengthened orthorhombic phase δ is incoherent with the γ matrix. Lenticular-shaped δ distributes in the FCC matrix. Lamellar-shaped δ nucleates at the grain boundary, which is used to control grain size in wrought material and also seems to be important for notch ductility [10,11,12]. Ultrasonic fatigue loading is an energy dissipated process, that accompanies a significant temperature increase for polyphase superalloys.

To date, few results have been presented in relation to the VHCF behavior and mechanism of Inconel 718. Zhong et al. [13] compared the HCF performance of solution aged and directly aged material using a high-frequency machine. Higher plasticity of the solid solution state material resulted in a large number of slip deformation zones, while the higher strength of this alloy made dislocation slip difficult after aged treatment. Esmaeilzadeha et al. [14,15] modified the model for predicting texture evolution. Kawagoishi’s team [16,17,18] demonstrated that fatigue strength increases with decreasing grain size. Moreover, the endurance limit under ultrasonic tension-compression is higher than that tested under rotary bending. Amanov et al. [19] presented that the improvement in fatigue life could mainly be attributed to induced compressive residual stress in the subsurface. Belan [8,9] found that Inconel 718 can still fracture after exceeding 10^8^ cycles. Both low and high cycle fatigue cracks initiate at large carbides whose length exceeds 50 μm. Yang et al. [20] indicated that the competition between surface and interior crack initiation behavior causes the inflection of the ***S-N*** curve. Manufacturing defects (e.g., gas pore, lack of fusion) in the microstructure could be the native micro-cracks resulting from the effective restriction. Texier et al. [21,22] investigated that the refinement in grain structure and higher Σ3 twin boundary density are associated with substantial reductions in lifetime. Pineau et al. [6] provided the modeling to predict minimum safe operating fatigue lives, dispersion associated with a variance in microstructure, and a component size effect. Effect of microstructure on the ultrasonic fatigue test remains unclear. The validity and accuracy of characterizing ultrasonic fatigue behavior in relation to self-heating phenomenon need to improve for a better understanding of this process in Inconel 718.

The present paper is focused on (1) evaluation of the microstructure induced by heat treatment, and (2) the influence of microstructure on the cyclic response and VHCF behavior of Inconel 718.

## 2. Experimental Procedures

**Figure 1:**
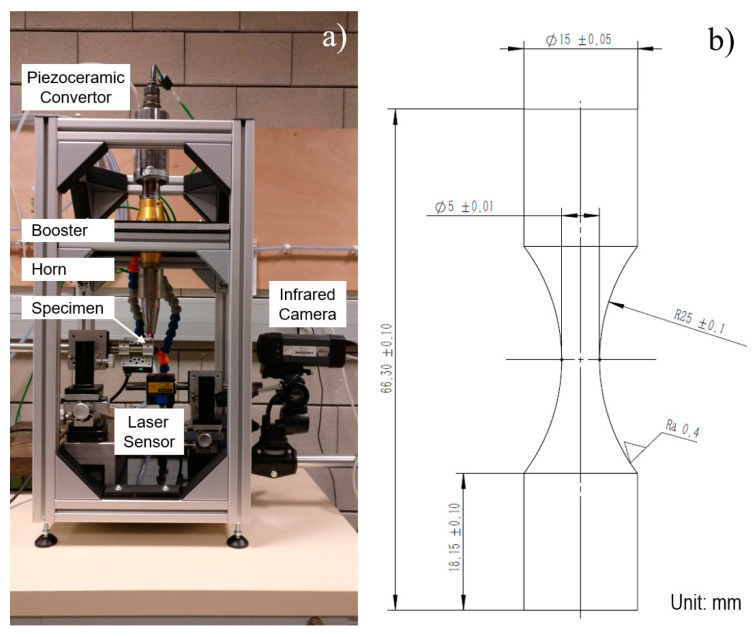
Ultrasonic fatigue test device and geometry of the Inconel 718 specimen: (**a**) schematic of device, (**b**) geometry of specimen.

Fully reversed tension compression loading (***R*** = −1) by the ultrasonic test system is studied in this test [23]. The schematics of the test device and specimen are shown in Figure 1. Four main parameters were monitored during the test. Resonance frequency was converted by the control voltage from the power generator. Temperature of the specimen was obtained from an infrared image of the test section by a Flir^®^ infrared camera (Wilsonville, OR, USA). When a crack propagates, surface temperature of the sample will increase suddenly due to the high level of thermal dissipation. Therefore, number of cycles at the crack initiation can be determined. Amplitude and mean value were calculated from the vibration wave on the top and bottom of the specimen and recorded using a Keyence^®^ laser sensor (Osaka, Japan) with a sampling rate of 200 kHz. Mean value was the relative distance between the probe and the detected surface, which was set at zero before the test. Specimen enlarged slightly due to the self-heating phenomenon, corresponding to the mean value increase. A self-programed data acquisition system by NI^®^ (Austin, TX, USA) LabVIEW was applied to conclude all the parameters. Fatigue test will be stopped artificially after 10^9^ cycles, even if the specimen has not broken yet.

To ensure resonance with the test device, the specimen was analytically designed and validated in a numerical way. The geometry of the specimen under VHCF vibrating fatigue is usually designed as an hourglass with axisymmetric profile, due to the larger stress amplification coefficient. Maximum stress stabilizes in the middle test section. Applied stress in the test section of the specimen presented a linear relationship with vibration amplitude, since the VHCF test was carried out under elastic condition.

## 3. Material Properties

### 3.1. Raw Material

Inconel 718 alloy (GH 4169 in China, NC19FeNb in Europe) is a high-strength, corrosion-resistant nickel-chromium material used at −250 to +700 °C. It can be fabricated even into complex parts, combining with good tensile, fatigue, creep, and rupture strength, which resulted in its use in a wide range of applications, for example in the components of aircraft and gas turbine engines, liquid-fueled rocket, and cryogenic tankage [24]. In this study, chemical composition of Inconel 718 was analyzed using Jeol^®^ energy dispersive X-ray spectroscopy (EDS, Tokyo, Japan), and the results are compared with manual results [24] in Table 1. The measured density of this sample batch of raw material is 8180.1 kg/m^3^.

### 3.2. Heat Treatment

Inconel 718 is typically purchased as annealed forging, billet, rod bar, plate, and stress-relieved conditions. This material is then fabricated in its most malleable condition. After fabrication, it could be heat-treated as required in terms of specifications according to the applicable specification.

For most applications, Inconel 718 is used in precipitation hardened (aged) condition, combining rupture life, rupture ductility, and impact strength, etc. This alloy is hardened by the precipitation of secondary phases (e.g., γ′ and γ″) into the metal matrix [21]. The specific process of aging treatment is as follows: solution at 720 °C for 8 h, then furnace cool to 620 °C at the rate of −50 °C/h for 2 h and continue aging for another 8 h at this temperature. The total treatment is 18 h followed by air cooling. The dynamic recrystallization (DRX) temperature is about 950 °C, according to the time-temperature-transformation diagram [25].

### 3.3. Microstructure

Metallographic analysis of Inconel 718 was investigated before and after heat treatment. Over 90% of fatigue life in metals is consumed in initiation in HCF and VHCF range at room temperature in general, which is highly influenced by the microstructure [1,5]. Leica^®^ DM ILM inverted light microscope (OM, Wetzlar, Germany) with Baumer^®^ TXG50c progressive scan camera (Friedberg, Germany) and Jeol^®^ JSM-6010Plus scanning electron microscope (SEM, Tokyo, Japan) were conducted to investigate the microstructure (phase, grain size, and precipitations).

The metallographic sample was sliced with an Al_2_O_3_ cut-off blade using a low feed speed of 0.01 mm/s. It was then polished with SiC emery paper of 80 to 2000 grit, and finished with 1/4 μm diamond paste for 30 min. All these steps were completed under water cooling to avoid increase in temperature. Afterwards, the sample was etched with homemade Kalling’s II reagent (also known as waterless Kalling) to reveal the grain boundary in less than 10 min after polishing, in order to avoid the passivity of the smooth surface.

**Figure 2:**
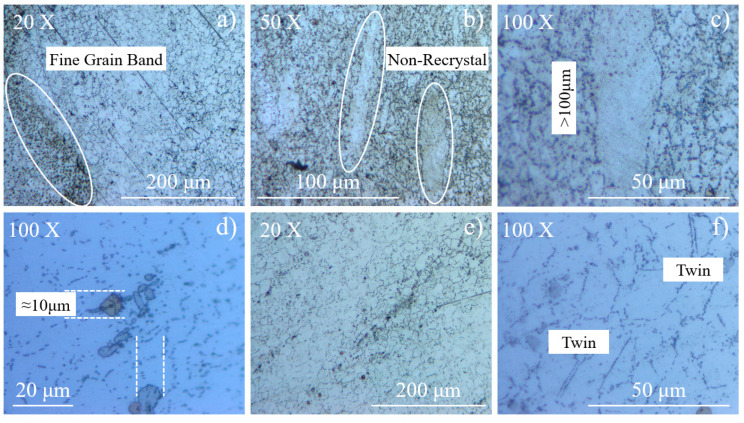
OM micrographs of annealed and aged Inconel 718: (**a**–**d**) annealed material, (**e**,**f**) aged material.

OM micrographs of Inconel 718 are shown in Figure 2. Heterogeneity can be seen in the microstructure of the annealed material. It contains a “fine grain band” (Figure 2a) consisting of a large amount of fine grains up to hundreds, and several “non-recrystallized” zones (Figure 2b). The length of these zones can exceed 100 μm, while the width is nearly 20 μm (Figure 2c). Size of precipitate particles is nearly 10 μm, close to the size of base γ grains (Figure 2d). “Fine grain band” and “non-recrystallized” grains disappear after aging treatment (Figure 2e). A twin boundary can still be found in the micrograph, with the length less than 10 μm (Figure 2f).

**Figure 3:**
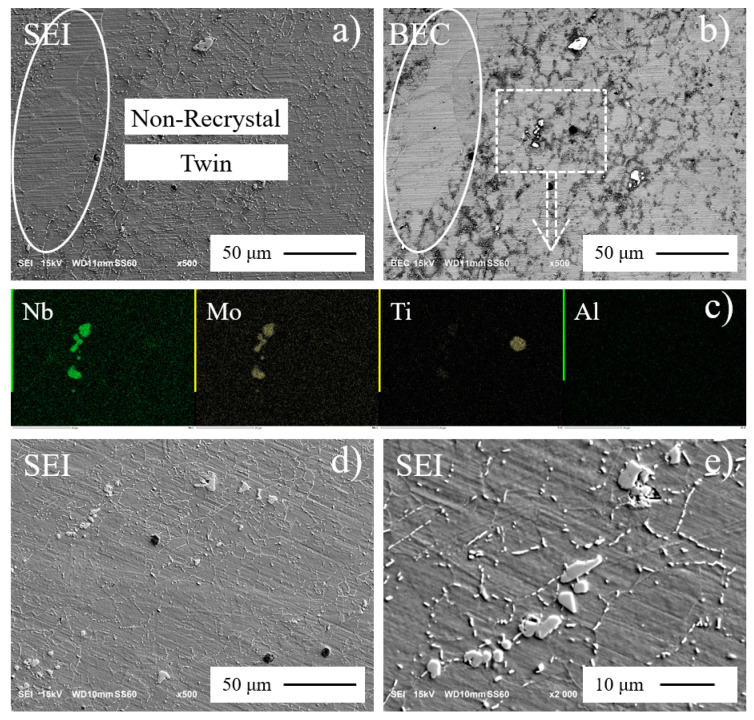
Scanning electron microscope (SEM) micrographs of annealed and aged Inconel 718: (**a**,**b**) SEM of annealed, (**c**) EDS of annealed, (**d**,**e**) SEM of aged material.

SEM micrographs and EDS maps of annealed and aged Inconel 718 are shown in Figure 3. Figure 3a,b show the same zone of annealed material under secondary electrons (SEI) and back-scattered electrons (BEC) modes. It is clear that the large non-recrystallized grains include several twins. The maximum twin boundary is nearly 50 μm, which seems to be the potential weakness initiation for fatigue failure.

The strength of superalloy Inconel 718 comes from coherent solid-state precipitates, which comprise small amounts of γ′ (Ni_3_Ti/Al), but are mostly γ″ (Ni_3_Nb), producing coherency strains in the γ FCC matrix. The γ′ has a unique morphology characterized by round particles that can be smaller than 200 Å, while γ″ is an ellipsoid with a length 5~6 times its thickness. The resolution of SEM is not sufficient to observe them, and transmission electron microscope (TEM) is needed to observe these morphologies [8,9]. Precipitate particles are lack of elements Ni, Cr, and Fe, which are the main elements of Inconel 718. The lighter particle is rich in Nb and Mo, and darker particle is rich in Ti and Al, as seen in Figure 3c.

Vickers micro-hardness tests with 25 g force loading and 10 s dwell time (HV_m_25/10) were used on grains and precipitations. Six independent measurements were applied and the results of 90% confidence intervals are listed in Table 2. The “Non-recrystallized” zone is softer than the “fine grain band” zone due to the lack of grain boundary. Precipitate particles are much harder than the basic matrix. Thermal expansion coefficients for inclusions or precipitations are far lower than the surrounding matrix. Apparent residual stress would develop in the boundaries during heat treatment [26]. These boundaries become another potential area for crack initiation. Fatigue life is reduced by several orders of magnitude when inclusion size increases for some superalloys [27].

“Fine grain band” and “non-recrystallized” grains disappear after aging treatment (Figure 3d). Non-strengthened orthorhombic phase δ (Ni_3_Nb) is incoherent with the γ matrix. Globularity and lenticular-shaped δ distributes in the FCC matrix. Needle-wafer-shaped δ nucleates at the grain boundary (Figure 3e). It is used to control grain size in wrought material and seems to be also important for notch ductility [28]. Grain boundary δ allows to identify the boundary between different grains, as well as the size of grains.

**Figure 4:**
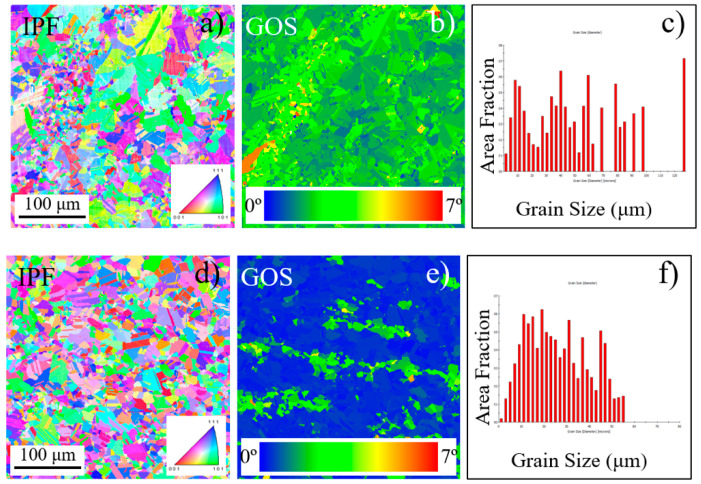
Electron back-scattered diffraction (EBSD) images of annealed and aged Inconel 718: (**a**–**c**) annealed material, (**d**–**f**) aged material.

Electron back-scattered diffraction (EBSD) images of annealed and aged Inconel 718 are shown in Figure 4. Grain orientation spread (GOS) is calculated as the average deviation of the orientation of each pixel in the grain from the average orientation for the grain [29]. Most of the grains with well-developed high-angle grain boundaries are characterized by no internal structure and uniform orientation. Several yellow and orange points appear in the center of the “fine grain band” zone (Figure 4b). This reveals that a high level of internal residual strain exists in these grains which are not sufficiently developed, becoming a potentially dangerous zone for fatigue crack initiation. For aged material, most areas are displayed in blue, since residual strain was relieved during aged treatment (Figure 4d). Histograms of grain size are shown in the images of Figure 4c,f. Grain diameter distributes in the range of 2~130 μm for annealed material, while all grains are smaller than 50 μm after aging treatment. The average is 24.6 μm, which is consistent with ASTM E112 grain size number 8 [30].

Directly aged high-quality (DAHQ) is a special type of Inconel 718 developed for advanced turbine discs. The δ phase not only exists in the grain boundary, but also diffuses into the base γ matrix in this edition of 718. The proportion and size of δ phase could be important factors determining fatigue behavior [21,31], which will be examined in a future study. In DAHQ Inconel 718, the grain diameter distribution is in the range of 4~10 μm, which is consistent with ASTM 10~12 [30]. Physical properties of these three materials are listed in Table 3.

## 4. Results

### 4.1. Monotonic Quasi-Static Uniaxial Tensile

The MTS^®^ Landmark™ 793 servo-hydraulic test system (Eden Prairie, MN, USA) with 647 hydraulic wedge grips was used to investigate the mechanical properties. The MTS^®^ 634.31F extensometer with a reference working length of $10_{-2}^{+4}$ mm was clamped to the calibrated section of the specimen for the simultaneous strain monitoring.

A monotonic quasi-static uniaxial tensile test under displacement control with loading speed of 0.05mm/min was carried out at room temperature (RT). The test followed the standard ASTM E8 [32]. The diameter of the test section is 6 mm, gauge length is four times, and reduced parallel section length is five times the diameter. Three samples were applied to each kind of material. Mechanical properties results are shown in Table 4. Good correlation can be noticed between tested values and from the Special Metal Corporation^®^ manual [24]. Raw Inconel 718 is typically purchased in an annealed high toughness condition in order to facilitate manufacture. Components could then be heat-treated as required after fabrication for high strength.

### 4.2. Cyclic Stress-Strain Response

Full reversed tension-compression testing under stress control (***R_σ_*** = −1) at 0.1 Hz was carried out using the MTS^®^ test system at RT. Four levels of loading were chosen in totally elastic (<σ_p_), slightly plastic (σ_p_~σ_0.2_, σ_0.2_ −50 MPa), and significantly plastic (σ_0.2_ + 50 MPa) conditions, respectively. The specific loading was determined depending on the quasi-static uniaxial tensile results from the last section.

**Figure 5:**
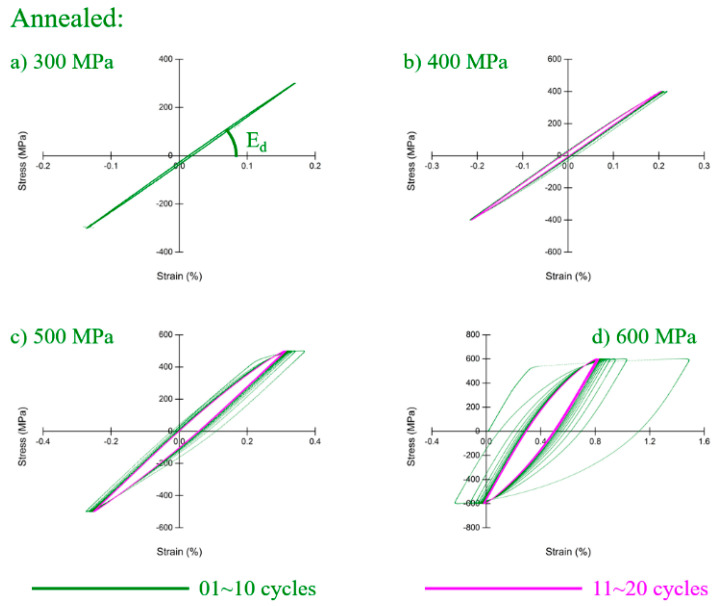
Hysteresis loop of annealed Inconel 718 (***σ*_0.2_** = 539 MPa): at (**a**) 300 MPa; (**b**) 400 MPa; (**c**) 500 MPa; (**d**) 600 MPa.

Cyclic response results for annealed Inconel 718 are shown in Figure 5. Totally elastic behavior occurred until 300 MPa (Figure 5a), and slightly plastic began to appear when loading reached 400 MPa (Figure 5b). A hysteresis loop developed from wide to narrow, and was constant after 20 cycles (Figure 5b–d). It indicates that the cyclic hardening phenomenon appeared in the annealed sample.

**Figure 6:**
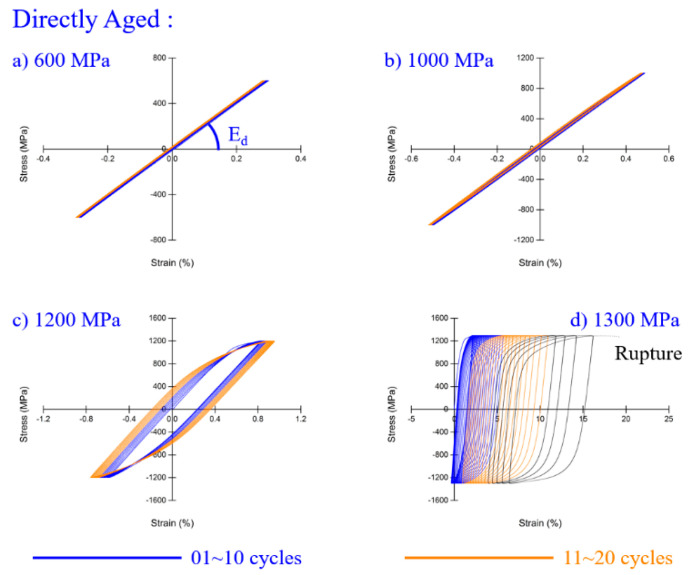
Hysteresis loop of aged Inconel 718 (***σ*_0.2_** = 1249 MPa): at (**a**) 600 MPa; (**b**) 1000 MPa; (**c**) 1200 MPa; (**d**) 1300 MPa.

Cyclic response results after aging treatment are shown in Figure 6. The material stayed elastic up to 1000 MPa, and turned plastic at loading of 1200 MPa. A hysteresis loop developed from narrow to wide, meaning that cyclic softening occurred for the aged material. There was a transition from cyclic hardening to cyclic softening after aged heat treatment. Cyclic behavior of metals is related to its yield ratio. Cyclic hardening always happens when the yield ratio is less than 0.7, while cyclic softening happens when it is greater than 0.8 [33]. The yield ratio changed from 0.56 to 0.86 after aging treatment, leading to the transition. The ratchetting effect and mean stress relaxation have an influence on the fatigue crack initiation life of the material [34].

The slope between the cyclic stress and strain relations in the total elastic linear range is the elastic modulus, and results are listed in Table 5. It increases slightly as the loading strain rate increases. Dynamic modulus of the annealed material at 20 kHz was obtained using the ultrasonic test machine.

### 4.3. Self-Heating Phenomenon

Ultrasonic fatigue is an energy dissipated process, that accompanies temperature variations on the specimen surface. Surface temperature increased rapidly after the test started and approached nearly stable distribution after a long enough time, in general, several minutes into the test.

**Figure 7:**
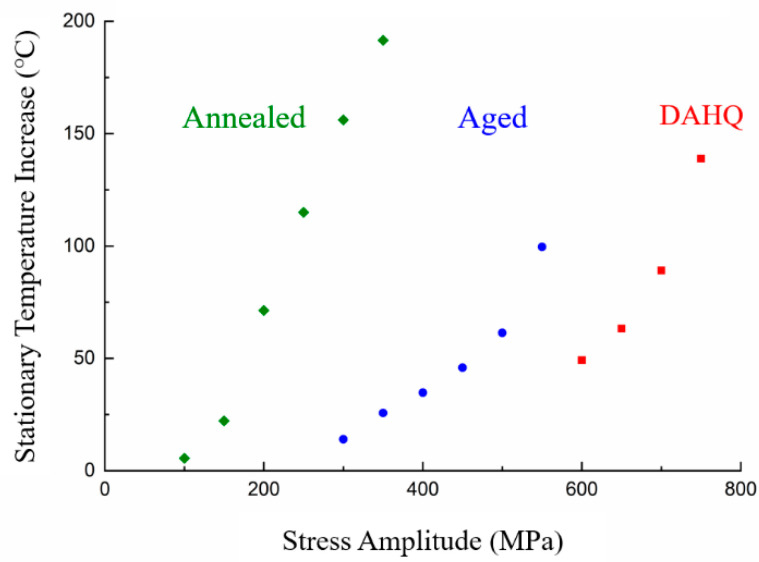
Stationary temperature increase of Inconel 718 (***R*** = −1, ***f*** = 20 kHz).

Maximum stationary temperature increase obtained from the specimen surface under different loading levels is shown in Figure 7. Energy dissipation is often related to the microstructure instability. Temperature increase associated with microstructural transformation seems to be the explanation for the frequency effects in ultrasonic fatigue tests [5]. It is clear that the self-heating phenomenon is attenuated by the homogenization process during aging treatment. It is assumed that the aged and DAHQ materials exhibit similar thermo-dynamic behavior, while the strength of the DAHQ sample is 200 MPa higher thanks to its fine grain.

Specimen temperature was far less than 700 °C, which is the design temperature limitation of Inconel 718. Therefore, the effect of the temperature increase on VHCF behavior can be treated as insignificant. It is necessary to point out that absolutely stable temperature distribution cannot be reached. Further sustained monitoring showed that temperature decreased 1~2 °C/h continuously for the annealed material, indicating that the cyclic hardening phenomenon continued to the VHCF regime. However, it increased slightly after aging treatment, implying the transition to cyclic softening.

### 4.4. Influence Factor on VHCF

**Figure 8:**
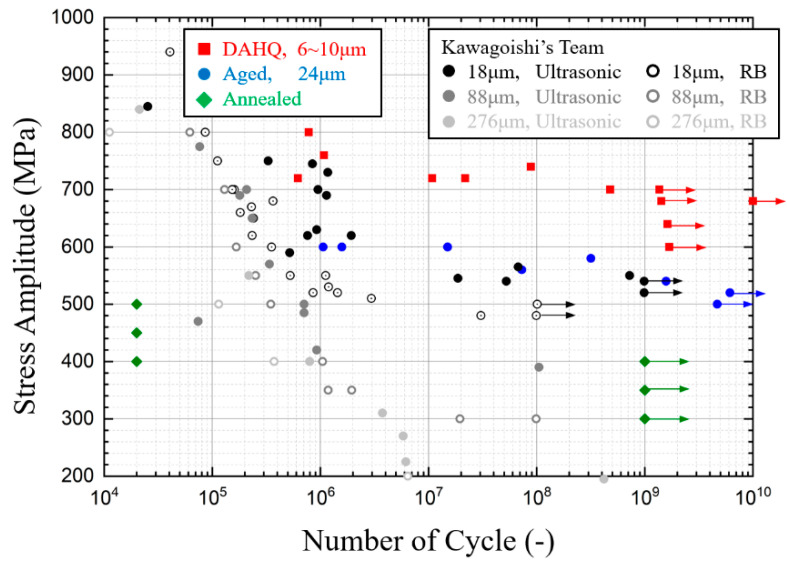
Comparison of VHCF properties with Kawagoishi’s team [16,17,18,37,38].

VHCF results for Inconel 718 under different heat treatment at 20 kHz are presented in the Appendix A Table A1 and are compared with other cases in the literature in Figure 8. The number of cycles corresponds to the total fatigue lifetime, which is counted from when the test device starts until when it stops due to the frequency loss when fracturing. Surface temperature recording allows determination of the transition from initiation to propagation due to the high elevation of thermal dissipation. Crack propagation duration continues from several seconds to tens of seconds depending on the total lifetime, while the duration is always less than 1% of total fatigue life.

Inconel 718 specimens still break after the traditional endurance limit of 10^8^ for non-ferrous metals. Fatigue strengths between 10^6^ and 10^9^ total life cycles have a difference of 100 MPa. Moreover, the comparison of DAHQ and aged data show a similar tendency, indicating the same mechanism for fracture. Fatigue strength for high-quality edition shows an improvement of 200 MPa from the common edition that we treated ourselves. The similar phenomenon can be found in the stationary temperature data in Figure 7.

However, for the annealed material, all specimens with loads greater than 400 MPa broke immediately, marked as 20,000 cycles (1 s) in Figure 8. Conversely, no specimens broke when loaded under 400 MPa. At the loading level of 400 MPa, several specimens broke immediately, while others never broke. It supposed that there is the threshold for small crack initiation, but its activation also depends on other conditions, such as microstructure or environmental effects. The ultrasonic fatigue test device at high frequency is unsuitable for annealed Inconel 718.

Kawagoishi’s team [16,17,18,35,36] from Japan investigated the influence of grain size on the fatigue properties of Inconel 718. Three sets of material with average grain size of 18, 88, and 276 μm were obtained by solution treatment at different temperatures followed by aging. Pulse-pause manner was applied during the test to minimize the temperature rise due to the internal friction of the material. For the material with larger grain size (in gray in Figure 8), the ***S-N*** curve is a straight line without inflexion. In other words, fatigue life increases continuously as loading decreases without a fatigue endurance limit. However, for the material with smaller average grain size of 18 μm (in black), it seems that there is a horizontal asymptote after sustained loading, 10^8^ cycles for rotary bending and 10^9^ for ultrasonic fatigue. It supposed that the average grain size significantly influences the fatigue properties. Another study also found that there is a transition in fatigue crack initiation mechanisms between fine-grained and coarse-grained GH 4169 at 15 Hz [37]. In the VHCF range between 10^7^ and 10^9^ cycles, the black data points by ultrasonic loading are really close to our aged material. VHCF strength is improved by grain refining. Pulse-pause mode, as well as specimen temperature control, are not decisive factors for superalloy Inconel 718, as long as the temperature increase is far lower than the design temperature limitation of 700 °C.

**Figure 9:**
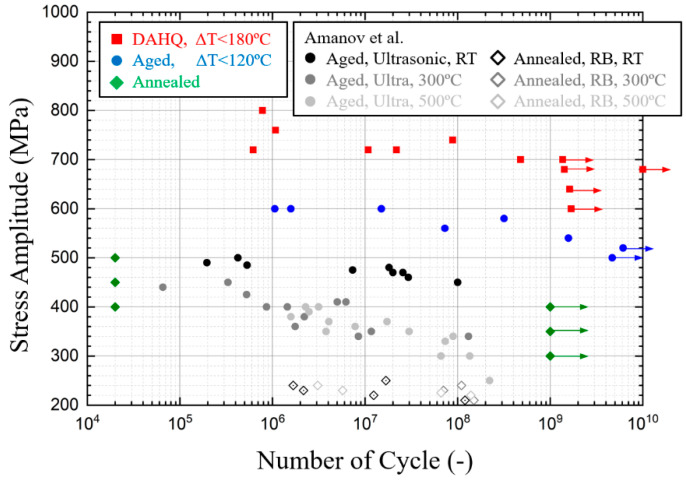
Comparison of VHCF properties with Amanov’s team [19].

Amanov [19] investigated the VHCF performance of Inconel 718 at RT and elevated temperature. The comparison of those results is shown in Figure 9. For the annealed material, fatigue strength is relatively lower than aged material, corresponding to the yield strength. Fast break is not reported under rotary bending (RB) test at 52.5 Hz, where there are fatigue fractures between 10^6^ and 10^8^ cycles. Relative to the RB test method, the fast break was not the natural property of the material but was induced by the high-frequency loading. So, the ultrasonic fatigue test method is not suitable for the annealed Inconel 718.

## 5. Discussion

Fracture surfaces were observed using OM and SEM, which revealed that specimens did not break into two parts automatically. The test group had stopped due to the resonance frequency decrease of the whole activation system, before the main crack was able to propagate until the whole test section. The cracked specimen was then pulled by another tensile fixture.

### 5.1. Annealed

**Figure 10:**
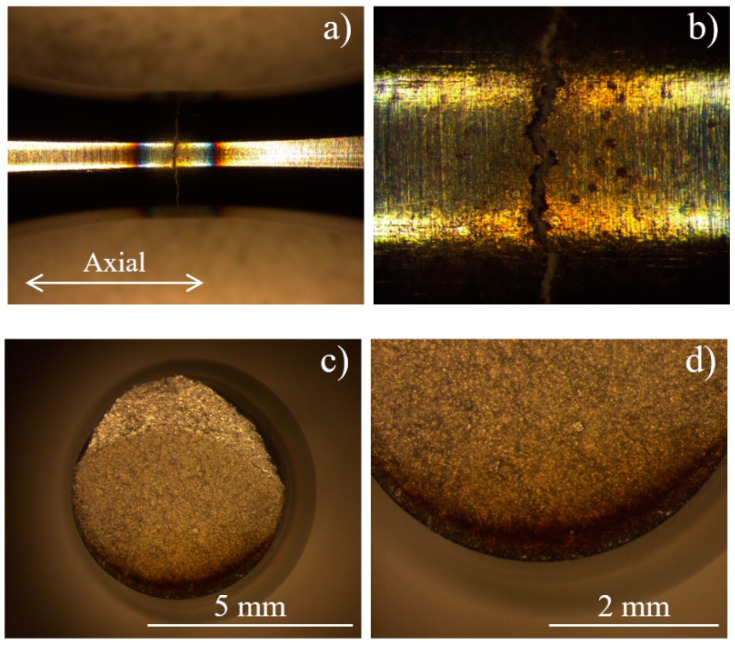
OM fracture images of annealed Inconel 718 (***R*** = −1, ***f*** = 20 kHz, ***σa*** = 400 MPa, ***Nf*** < 2 × 10^4^ cycles): (**a**,**b**) specimen surface; (**c**,**d**) fatigue fracture.

As mentioned before, some annealed specimens broke immediately under 400 MPa loading. OM fracture images of annealed material are shown in Figure 10. This is not a typical fatigue fracture, because there is no characteristic radial stair marks or circumferential striations, which appear generally in the fatigue fracture. There is a burn ring around the crack, indicating that a high-temperature gradient exists in the section (Figure 10a). Numerous macroscopic “holes” exist close to the crack, which is found in the highest temperature zone, maybe due to the dropping of grains cluster at the surface during the test (Figure 10b). It supposed that the main crack originated from these holes. Axial fracture surface images validate this point, the dark edge is much lower than the other plane, due to the grains block dropping (Figure 10c,d). Ultrasonic high-frequency loading significantly accelerates the small crack growth [38]. Regardless of the cause, once nucleation of a micro-crack has been activated, the crack will immediately propagate, leading to catastrophic breakage of the specimen.

**Figure 11:**
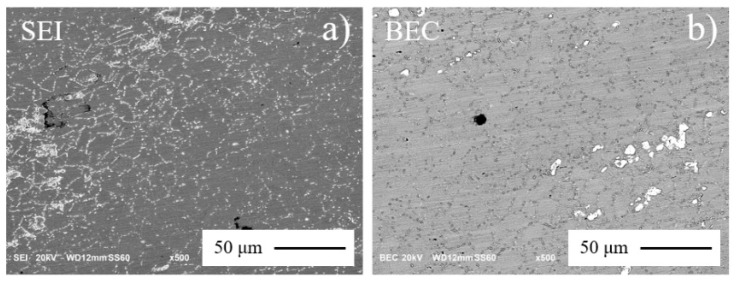
SEM micrographs of annealed Inconel 718 after (***R*** = −1, ***f*** = 20 kHz, ***σa*** = 400 MPa, ***Nf*** > 1 × 10^9^ cycles): (**a**) SEI mode; (**b**) BEC mode.

However, the other specimens did not break under 400 MPa until 10^9^ life cycles had been completed. The uncracked specimen was cut at the center of the test section using the same process as for the metallographic sample. Ultra-low cutting and polishing speed with water cooling ensured that no additional changes were introduced into the surface. SEM micrographs are shown in Figure 11. Similar to the aged metallographic micrographs, the “fine grain band” and “non-recrystallized zone” disappeared after cyclic tension-compression. Heterogeneous microstructure was ameliorated by ultrasonic loading. Self-heating during the fatigue process is always associated with a microstructural transformation [5]. It supposed that dynamic recrystallization has occurred, even if the test temperature had not reached the dynamic recrystallization (DRX) temperature of 950 °C [25].

### 5.2. Aged

**Figure 12:**
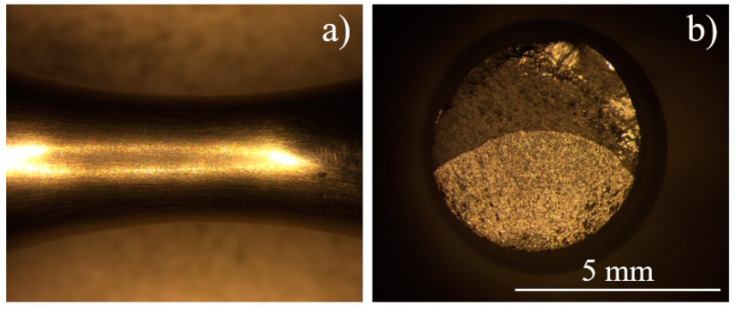
OM fracture images of aged Inconel 718 (***R*** = −1, ***f*** = 20 kHz, ***σa*** = 540 MPa, ***Nf*** = 1.57 × 10^9^ cycles): (**a**,**b**) specimen surface.

OM fracture surface images of aged Inconel 718 are shown in Figure 12. In general, one single initiation site exists regardless of loading level and fatigue life. “Fish-eye”, which occurs in some cases of VHCF [39,40], does not appear in the fracture. Initiation is situated at the surface or close to the subsurface. The propagation duration distributes from several seconds to tens of seconds depending on the total life, and always occupies less than 1% of the total fatigue lifetime.

**Figure 13:**
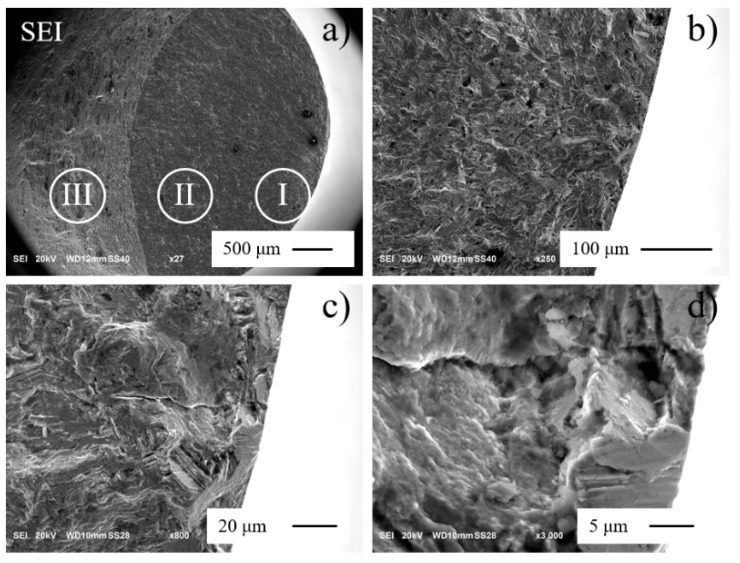
SEM fracture images of aged Inconel 718 (***R*** = −1, ***f*** = 20 kHz, ***σa*** = 540 MPa, ***Nf*** = 1.57 × 10^9^ cycles): (**a**) fatigue fracture, (**b**) initiation-low zoom; (**c**) secondary crack, (**d**) initiation-high zoom.

SEM micrographs of fracture surface of aged Inconel 718 are shown in Figure 13. Fatigue fracture can generally be divided into several typical areas (Figure 13a): initiation area I, with a relatively flat surface, propagation area II, with a distinctly rough area and with radial streaks along the crack propagation direction, and dimples zone III due to overload. Fine granular area (FGA) [41] indicates the boundary of initiation area I. A secondary crack perpendicular to the main crack significantly improves the fatigue life (Figure 13b,c). There is no obvious inclusion in the nucleation zone. It assumed that the crack originates from the slip bands in the direction of the maximum shear stress (Figure 13d). 

## 6. Conclusions

(1) Three sets of materials of Inconel 718 with different heat treatment methods, namely annealed, aged, and directly aged high-quality edition (DAHQ) were compared. Differences in grain size, phase, and precipitate particles were investigated using a metallographic micrograph by OM and SEM. Heterogeneity, such as in the form of “fine grain band” and “non-recrystallized grains”, was reduced and residual strain was also relieved after aged treatment.
(2) Quasi-static uniaxial tensile properties and cyclic response of Inconel 718 were proposed. Yield and ultimate strength increased obviously after aging heat treatment. Self-heating phenomenon at 20 kHz was attenuated by heat treatment as grain size decreased. There was a transition from cyclic hardening to softening after aging heat treatment.
(3) The crack initiation duration occupied greater than 99% of the total fatigue life for Inconel 718 at 20 kHz. Average grain size influenced the VHCF strength and the crack initiation mechanism. Self-heating phenomenon is not a decisive factor influencing VHCF strength of superalloy Inconel 718, as long as the temperature increase is far less than the design temperature limitation of 700 °C.
(4) Heterogeneous microstructure was ameliorated by ultrasonic loading. The “Fine grain band” and “non-recrystallized zone” disappeared after cyclic tension-compression. A single initiation site was presented in the aged specimen, regardless of the loading level and total fatigue life cycles. Micro-crack initiated at subsurface slip bands in the direction of maximum shear stress.

## Figures and Tables

**Table 1:** Limiting chemical composition (Mass%).

Element	Ni	Cr	Fe	Nb	Mo	Ti	Al
Manual	50~55	17~21	Bal	4.75~5.5	2.8~3.3	0.65~1.2	0.2~0.8
EDS	51.10	19.62	20.28	4.78	2.52	1.14	0.56

**Table 2:** Micro-hardness of different phases in annealed Inconel 718.

	Non-Recrystallized Zone	Fine Grain Band	Dark Precipitation	Light Precipitation
HVm25/10	269 ± 14	319 ± 18	524 ± 36	>800

**Table 3:** Physical properties of Inconel 718 before and after heat treatment.

	Hardness (HRC)	Grain Distribution (μm)	Mean (μm)	Standard Deviation (μm)	ASTM (-)
Annealed	22	2~130	51.1	34.2	N/A ^#^
Aged	43	2~50	24.6	13.6	8
DAHQ	46	4~10	N/A ^##^	N/A ^##^	10~12

^#^ not available for heterogeneous grain; ^##^ confidential.

**Table 4:** Mechanical properties before and after heat treatment.

	E (GPa)	*σ*_0.2_ (MPa)	σ_UTS_ (MPa)	δ/A (%)	ψ/Z (%)
Annealed	190.6	539	957	58	49
Aged	201.5	1249	1456	29	28

**Table 5:** Dynamic modulus under different loading frequencies.

Elastic Modulus (GPa)	Quasi-Static	0.1 Hz	20 kHz
Annealed	190.6	200.7	207.3
Aged	201.5	209.7	N/A ^#^

# not tested.

## Appendix A

**Table A1:** Relationship between applied stress and number of cycles tested at 20 kHz.

	DAHQ			Aged	
Stress	Cycle	Comment	Stress	Cycles	Comment
800	7.80 × 10^5^		600	1.06 × 10^6^	
760	1.08 × 10^6^		600	1.58 × 10^6^	
740	8.84 × 10^7^		600	1.50 × 10^7^	
720	6.20 × 10^5^		580	3.17 × 10^8^	
720	1.08 × 10^7^		560	7.28 × 10^7^	
720	2.18 × 10^7^		540	1.57 × 10^9^	
700	4.78 × 10^8^		520	6.13 × 10^9^	run out
700	1.36 × 10^9^	run out	500	4.68 × 10^9^	run out
680	1.42 × 10^9^	run out			
680	1.0 × 10^10^	run out			
640	1.62 × 10^9^	run out			
600	1.68 × 10^9^	run out			
